# Acute Pancreatitis Simulating Myocardial Infarction: A Challenging Case

**DOI:** 10.7759/cureus.37769

**Published:** 2023-04-18

**Authors:** Saher T Shiza, Aalok Parajuli, Iqra Samreen, Tripura Padullaparthi, Alaa S Mohamed, Muhammad Haseeb, Haleema Sadia, Khalid H Mohamed, Hira Nasir

**Affiliations:** 1 Internal Medicine, New York City Health and Hospitals - Lincoln Hospital, New York, USA; 2 Internal Medicine, Jalalabad Ragib Rabeya Medical College & Hospital, Jalalabad, BGD; 3 Internal Medicine, Deccan College of Medical Sciences, Hyderabad, IND; 4 Internal Medicine, Malla Reddy Medical College for Women, Hyderabad, IND; 5 Neurology, Augusta University, Augusta, USA; 6 Internal Medicine, Allama Iqbal Medical College, Lahore, PAK; 7 Internal Medicine, Khyber Teaching Hospital Peshawar, Peshawar, PAK; 8 Neurology, Sheffield Teaching Hospitals NHS Foundation Trust, Sheffield, GBR; 9 Internal Medicine, Mayo Hospital, Lahore, PAK

**Keywords:** mi, acute coronary syndrome, myocardial infarction, acute pancreatitis complication, acute pancreatitis

## Abstract

Acute pancreatitis is an inflammatory condition with varying local and systemic complications and variable severity. Although rare, cardiovascular complications induced by acute pancreatitis are rarely described in the literature. Epigastric pain with acute pancreatitis often simulates electrocardiographic changes in the absence of coronary artery abnormalities, resulting in a diagnostic dilemma for optimal treatment and management. We underline a case of acute pancreatitis complicated by acute coronary syndrome in a patient who presented with chest heaviness, dyspnea, nausea, and worsening epigastric pain associated with vomiting. Clinical and laboratory evaluations and using imaging modalities were suggestive of acute pancreatitis mimicking myocardial infarction (MI) in the absence of coronary artery abnormalities.

## Introduction

Acute pancreatitis is a condition characterized by inflammation of the pancreas and may present with severe abdominal pain, nausea, and vomiting. Acute pancreatitis is usually self-limiting but may progress into severe pancreatitis with systemic and local complications [1]. Complications include systemic inflammatory response syndrome, infection, renal failure, and cardiovascular complications. Hypovolemia, shock with the systemic inflammatory syndrome, pericardial effusion, and non-specific ST segment changes are among the cardiovascular complications of acute pancreatitis [2]. Acute pancreatitis mimicking myocardial infarction (MI) is not widely highlighted in the literature [3]. Here, we report a case of acute pancreatitis complicated by MI, causing chest symptoms in the absence of coronary artery abnormalities.

## Case presentation

A 52-year-old male patient presented to the emergency department complaining of severe chest pain and shortness of breath. The patient reported that he had been experiencing intermittent epigastric pain for the past two days, which had worsened in the last 12 hours. He also reported nausea, vomiting, and diarrhea. The patient denied any past medical history, surgical history, or medication use. However, he admitted to drinking alcohol daily for the past 20 years. Upon arrival, his vital signs were as follows: blood pressure 100/60 mmHg, heart rate 110 bpm, respiratory rate 22 bpm, and oxygen saturation 95% on room air. The patient was in distress and appeared anxious.

On physical examination, he appeared to be in moderate distress, tachypneic, with shallow breathing and using accessory muscles. Chest auscultation revealed clear lung fields and cardiovascular examination showed tachycardia with a regular rhythm. Epigastric tenderness was noted on palpation, with guarding and rebound tenderness. Bowel sounds were diminished, and there was no palpable mass or hepatosplenomegaly.

**Figure 1:**
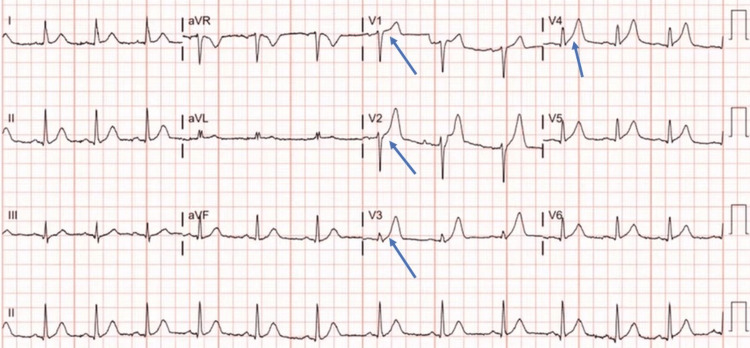
Electrocardiography demonstrating ST-segment elevation and hyperacute T wave in leads V1-V4.

**Figure 2:**
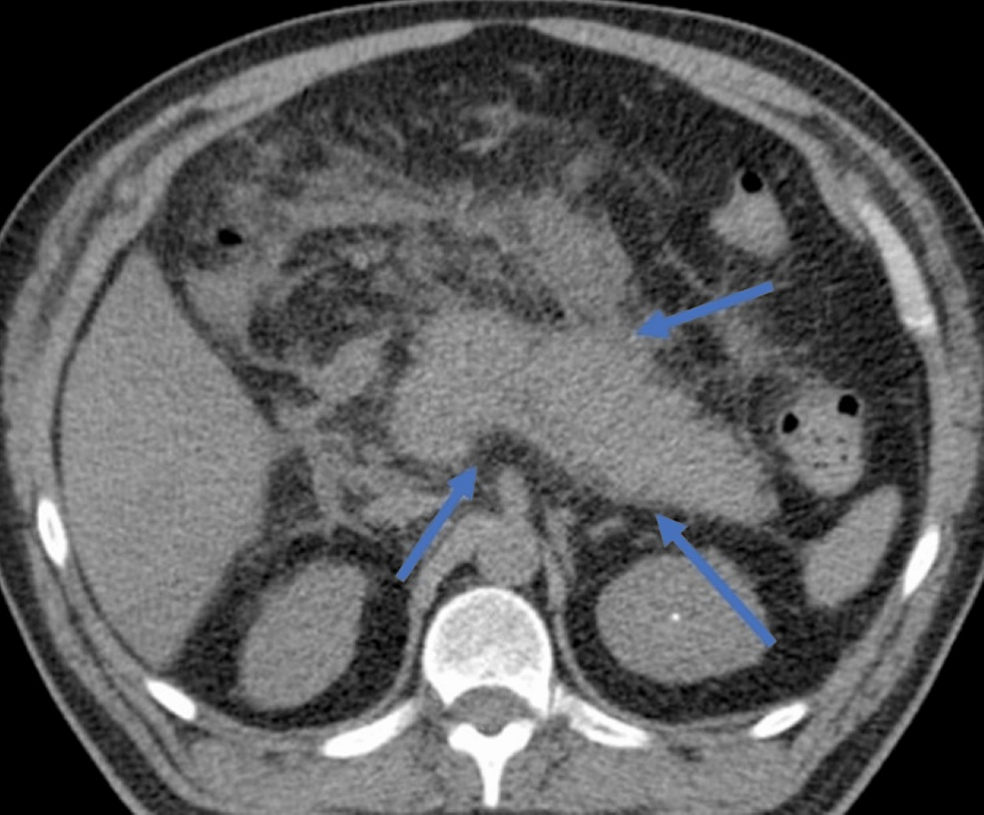
Abdominal computed tomography revealing diffuse pancreatic enhancement with fat stranding and peripancreatic fluid (blue arrows).

An electrocardiogram (EKG) was performed, which showed sinus tachycardia, ST-segment elevation in leads V1-V5, and hyperacute T waves in the same leads, suggestive of acute MI (Figure 1). The troponin I level was 2.5 ng/mL (0-0.04). He was immediately started on dual antiplatelet therapy with aspirin and clopidogrel, and heparin was administered as well. He was transferred to the cardiac catheterization lab for urgent angiography. However, his condition deteriorated, and he became hypotensive, with a blood pressure of 80/40 mmHg and tachycardia of 130 bpm. An abdominal computed tomography (CT) was performed, which revealed signs of acute pancreatitis, including diffuse enhancement, pancreatic edema, and peripancreatic fluid (Figure 2). Serum lipase and amylase (580 IU/L) were found to be elevated (Table 1). Echocardiography showed inferoseptal hypokinesia with a left ventricular ejection fraction estimated at 45%.

**Table 1:** The results of laboratory evaluations.

Parameter	Lab value	Reference range
Amylase	580 IU/L	30-110
Lipase	980 IU/L	0-155
Creatinine	1.9 mg/dl	0.7-1.3
Blood urea nitrogen	27 mg/dl	08-26
White cell count	9100 /mm^3^	4000-11000
Hemoglobin	12 g/dl	14-16
Erythrocyte sedimentation rate	17/hour	<22
Alkaline phosphatase	85 mg/dl	35-95
Alanine aminotransferase	45 IU/L	8-56
Calcium	9.4 mg/dl	9.0-10

He was immediately shifted to the intensive care unit for further management and was commenced on aggressive fluid resuscitation with intravenous (IV) fluids, bowel rest, supportive care, and electrolyte replacement. Pain control was achieved with IV opioids, and antiemetic therapy was initiated.

**Figure 3:**
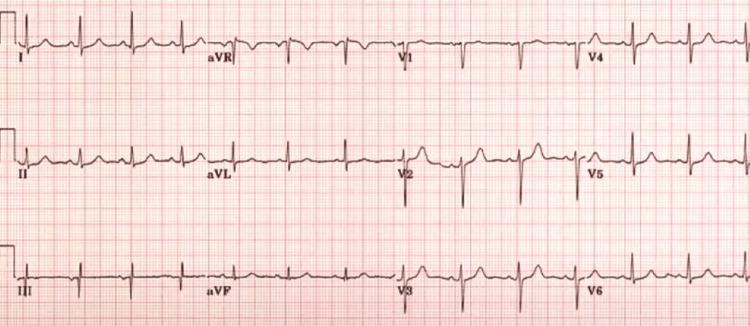
Repeat EKG on admission day six. EKG: electrocardiogram.

**Figure 4:**
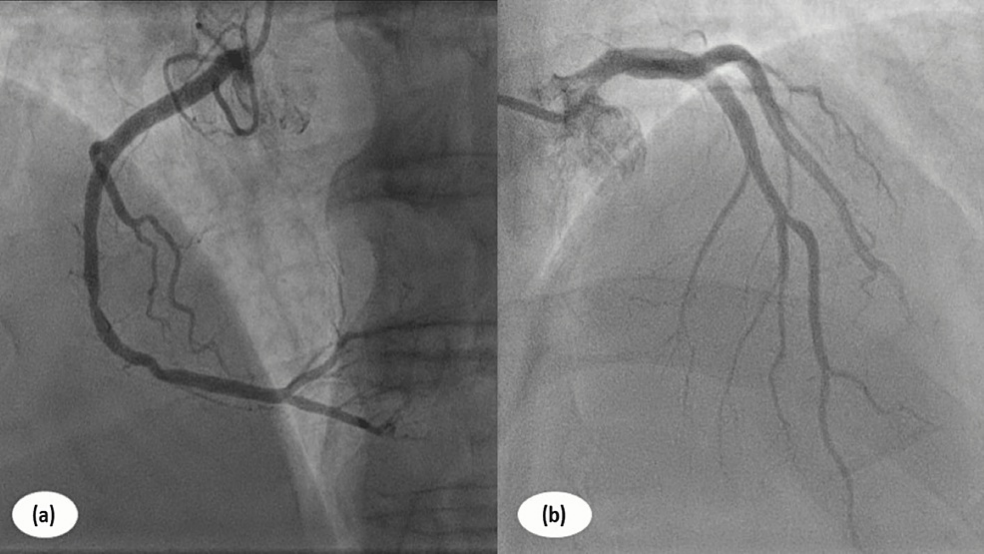
Coronary angiography demonstrating normal right (a) and left (b) coronary artery and associated branches.

Over the next few days, his condition improved, with a decline in the severity of abdominal pain and normalization of his vital signs. Repeat laboratory investigations showed a decline in serum amylase and lipase levels, as well as an improvement in renal function. Cardiac MRI was not performed due to a lack of facilities. A repeat ECG was performed, which showed normalization of ST-segment elevation, and the Troponin I level decreased to 1.2 ng/mL (Figure 3). A follow-up coronary angiogram revealed no significant coronary artery disease with normal ventricular ejection fraction on repeat echocardiography (Figure 4).

## Discussion

Acute pancreatitis can progress into severe acute pancreatitis with local and systemic complications. Local complications of acute pancreatitis include pancreatic pseudocyst, pancreatic necrosis, and pancreatic abscess. Systemic complications of acute pancreatitis include respiratory failure, kidney failure, acute lung injury, acute respiratory distress syndrome, and sepsis [2]. Although rare, cardiovascular complications can also occur in patients with acute pancreatitis, which include hypovolemia, pericardial effusion, shock or worsening of underlying ischemic heart disease, or heart failure [4]. Rarely acute pancreatitis can also be associated with many EKG changes, including arrhythmias and changes in T wave and ST-segment elevation mimicking myocardial infarction, which is not widely reported in the literature [5-10]. We have tabulated acute pancreatitis-induced cardiovascular complications in Table 2.

**Table 2:** Reported cases of acute pancreatitis causing myocardial infarction. M: male, F: female, EKG: electrocardiogram

Author et al.	Age/Sex	Clinical presentation	Lab values	ECG changes	Antithrombotic administration	Outcome
Mouedder et al [5].	63/F	Chest pain, vomiting	Elevated lipase and troponin I	ST-elevation in posterior leads	Yes	Discharged
Egashira et al. [6]	31/F	Fever, abdominal pain	Elevated lipase and troponin level	ST-elevation in anterolateral leads	No	Improved with resolution of EKG changes
Khan et al. [7]	30/F	Vomiting, abdominal pain, chest pain	Elevated troponin T and amylase	ST-elevation in anterolateral leads	Yes	Discharged, EKG changes resolved
Hajimoradi et al. [8]	35/M	Epigastric pain, vomiting	Elevated lipase, amylase	ST-elevation in inferior and anterolateral leads	Yes	Discharged, EKG changes resolved
Agrawal et al. [9]	60/M	Syncope	Elevated lipase	ST-elevation in inferior and anterior leads, peaked T waves	No	Discharged, persistence of EKG changes.
Vasantha et al. [10]	36/M	Epigastric and retrosternal chest pain	Elevated lipase, amylase,	ST-elevation in septal leads	Yes	Discharged, resolution of EKG changes
Long et al. [11]	63/F	Abdominal pain, vomiting	Elevated lipase, amylase, troponin T	ST-elevation in inferior leads	Yes	Complicated by multi-organ failure
Ralapanawa et al. [12]	58/F	Vomiting, epigastric pain	Elevated amylase, lipase, troponin	Lateral ischemia	Yes	Discharged with persistence of EKG changes

The pathophysiology of acute pancreatitis-induced MI is complex and not fully understood. It is thought to involve a combination of direct and indirect effects on the heart, including damage to the blood vessels, hypercoagulability, and changes in cardiac function [13]. Several risk factors can increase the likelihood of developing AMI in this setting, which include advanced age, any previous heart disease, and a history of smoking [8]. Acute pancreatitis mimics acute myocardial infarction and is typically due to electrification of the autonomic nervous system, especially the sympathetic nervous system, which can cause multiple cardiac manifestations [14]. The sympathetic nervous system is activated in response to pain and stress and can augment tachypnea, tachycardia, blood pressure elevation, and increased cardiac output. This can lead to symptoms that are similar to those seen in acute myocardial infarction, such as chest pain and shortness. Cardiac involvement in acute pancreatitis may also be due to stress-causing catecholamine discharge, which is part of Takotsubo syndrome or stress cardiomyopathy, preceded by an emotional, physical, or combined trigger, releases an excess of catecholamines that are thought to be the cause of myocardial kinetic disorders.

Due to the rarity of acute pancreatitis-associated MI, there is no standardized management protocol. Identifying underlying mechanisms is crucial due to various etiologic factors that can result in ST elevation. Differentiating between real and pseudo-MI is mandatory as the management strategies vary greatly for both conditions. Using thrombolytics for pseudo-MI misdiagnosed as real may lead to severe bleeding, particularly when there is underlying acute pancreatitis [4-6]. In patients with acute pancreatitis, symptoms like MI, troponin, brain natriuretic peptide levels, and electrolyte imbalances must be addressed, especially hyperkalemia which causes ST-segment changes [13]. Echocardiography can be performed in stress cardiomyopathy with elevated cardiac markers. When there is a high risk of acute coronary syndrome, coronary angiography can also be advised after evaluating the risk, benefits, and need for antithrombotic use [11-14]. In certain cases, conservative management with lipid-lowering drugs and antithrombotics may be advised, as acute pancreatitis is an absolute contraindication to coronary intervention [6]. In general, it is essential to keep in mind that diagnosis of pseudo-MI in acute pancreatitis or EKG changes associated with pancreatitis is the diagnosis of exclusion, and coronary artery disease must be ruled out even in the presence of chest pain or heaviness.

Our patient presented with chest heaviness, dyspnea, nausea, and worsening epigastric pain associated with vomiting. His clinical picture, laboratory evaluations, and electrocardiographic changes with rapid recovery of ventricular function on echocardiography and normal angiography were suggestive of acute pancreatitis mimicking acute non-ischemic MI in the absence of coronary artery abnormalities.

## Conclusions

Acute pancreatitis can be associated with electrical modifications mimicking acute coronary syndrome in the absence of coronary artery abnormalities. Acute pancreatitis mimicking MI is a rare but critical condition that requires careful evaluation and management. The similarities in presentation between these two conditions can make diagnosis challenging, but a high degree of suspicion and cautious assessment can help distinguish between them. Early and accurate diagnosis is crucial to ensure appropriate management and prevent potential complications. The underlying mechanism of this association remains undefined and further studies are warranted to explain this challenging condition.
